# Determination of minimal transcriptional signatures of compounds for target prediction

**DOI:** 10.1186/1687-4153-2012-2

**Published:** 2012-05-10

**Authors:** Florian Nigsch, Janna Hutz, Ben Cornett, Douglas W Selinger, Gregory McAllister, Somnath Bandyopadhyay, Joseph Loureiro, Jeremy L Jenkins

**Affiliations:** 1 Developmental and Molecular Pathways, Novartis Institutes for BioMedical Research, Forum 1, Novartis Campus Basel, CH-4056, Basel, Switzerland; 2 Developmental and Molecular Pathways, Novartis Institutes for BioMedical Research, 220 Massachusetts Avenue, 02139 Cambridge, MA, USA; 3 Immunology Clinical Biomarkers, Bristol Myers Squibb, Princeton, New Jersey

**Keywords:** transcriptional profiling, target prediction, genetic algorithm, graphics processing unit (GPU) programming, compute unified device architecture (CUDA)

## Abstract

The identification of molecular target and mechanism of action of compounds is a key hurdle in drug discovery. Multiplexed techniques for bead-based expression profiling allow the measurement of transcriptional signatures of compound-treated cells in high-throughput mode. Such profiles can be used to gain insight into compounds' mode of action and the protein targets they are modulating. Through the proxy of target prediction from such gene signatures we explored important aspects of the use of transcriptional profiles to capture biological variability of perturbed cellular assays. We found that signatures derived from expression data and signatures derived from biological interaction networks performed equally well, and we showed that gene signatures can be optimised using a genetic algorithm. Gene signatures of approximately 128 genes seemed to be most generic, capturing a maximum of the perturbation inflicted on cells through compound treatment. Moreover, we found evidence for oxidative phosphorylation to be one of the most general ways to capture compound perturbation.

## Introduction

Early drug discovery research involves target discovery and lead discovery. Target discovery is concerned with the identification and validation of the disease-relevance of a particular protein. Subsequent lead discovery is the task of finding a suitable molecule that can interact with the target in a specific, therapeutically relevant way. A typical strategy to identify potential lead compounds is the screening of large collections of molecules, up to several millions, in highly automated high-throughput assays. In biochemical assays, each molecule is tested against a purified target protein of interest; molecules that are found to significantly affect the assay readout are called hits and are selected for further follow-up experiments such as secondary- or counter-screens. Successful outcomes in those latter screens result in more confidence of having found a true modulator of the target protein, yielding a target-lead pair. An orthogonal approach where the target protein is unknown from the outset is a phenotypic screen: a collection of molecules is tested for their potential to induce (or abrogate) a complex phenotype, such as the ability of cells to divide successfully. Because the target protein of such screens is not known, they require the identification of the target that gives rise to the observed phenotype subsequent to the identification of active compounds.

Whereas biochemical assays have the advantage that the target protein is essentially a parameter of the experiment, they often lack biological relevance because compounds tested do not have to penetrate cell walls and are not subjected to other relevant biological processes such as active transport and metabolism. Phenotypic assays are a more realistic model for compound administration to living systems but entail the significant post-screen difficulty of target identification and mode of action (MoA) elucidation for any hits identified.

The identification of molecular target and MoA of compounds is a key hurdle in drug discovery. Significantly more hits are obtained from screening campaigns than are typically amenable to extensive experimental profiling such as proteomics. Computational methods that inform about the underlying, specific biological processes, for example targets and pathways, that are actually being perturbed by the compounds are much sought after, as they can help to uncover the molecular causes of the positive assay readout. Many such methods rely on the availability of compound annotations from previous experiments or specific profiling platforms. There is a considerable amount of literature on target prediction methods that work from chemical structure alone or composite data types using a variety of methods, and we refer the interested reader to [1-4] and the references therein. Profiling platforms are composed of a reference base of *n*-dimensional readouts, for example a panel of reporter gene assays [5], for a set of well-characterised compounds and a mechanism to position the readouts of novel samples in the context of the reference. This latter mechanism is often some kind of metric such as Euclidean distance or Pearson correlation, though more sophisticated methods can also be applied.

Transcriptional profiles, the mRNA levels of expressed genes as a result of treatment of cells with a compound, are routinely used to cluster or otherwise relate compounds that elicit a similar biological response [6-8]. For any such approach, it is important to choose which genes to include in the calculations. Typical human genome-wide chips cover approximately 22,000 genes, where the expression level of each gene is determined by a set of specific probes, a probeset [9]. Other experimental techniques, however, require the selection of a set of genes upfront, for example the Luminex technology of Panomics [10]. The selection of suitable genes, a gene signature, depends on the desired signature size, which is directly proportional to cost, as well as the biological questions that need to be addressed. The selection and evaluation of such gene signatures is the subject of the remainder of this article. Like many other companies, Novartis has several compound profiling platforms, including one based on expression profiles. The questions that we addressed in this article are directly related to some of our ongoing efforts to optimise such platforms.

We used a publicly available microarray dataset [7] in conjunction with extensive compound annotations to probe several important aspects of target and MoA prediction from gene signatures. We explored systematically to what extent transcriptional profiles of compounds can be used for target prediction. This study provided insight into questions such as the following: Is there and what is the minimal gene signature that can be used to reasonably predict molecular targets of compounds? Do designed signatures predict targets better than genes selected at random? How can such signatures be optimised in an automatic way, and what are the results of such an optimisation? We employed machine learning and biologically inspired algorithms implemented on state-of-the-art graphics processing units (GPUs) to answer these questions.

## Results and discussion

### Compound-target annotations

We retrieved all currently known targets for any compound in Connectivity Map 2 [7] where the compound had an activity (IC_50_, *K_i_*) of ≤ 5 μM. Each compound had an average number of 23 targets satisfying these criteria. The compound with the most targets was staurosporine with 386, whereas for 126 molecules only one target was known, for example hydroxysteroid (17-beta) dehydrogenase 1 (HSD17B1) for the horse steroid equilin. At least 5 targets were known for 502 compounds. These high numbers of high-affinity targets per compound illustrate the fact that many compounds, including many marketed drugs [4,11], are much less specific than is typically appreciated. A further compounding factor for this polypharmacology comes from the tissue expression of the drug targets. A compound with several high-affinity *in vitro *targets could not manifest its action at all of these proteins if most of them were not expressed. The tissue expression of many proteins, however, is relatively unspecific: recent RNA-sequencing experiments showed that approximately 6,000 genes were expressed in all of heart, liver, testis, skeletal muscle and cerebellum, all of which are important target tissues for therapeutics [12,13]. Targeted drug delivery and carefully designed pharmacokinetic compound properties can provide some relief; yet, it is obvious that the foundations for polypharmacology have been laid in evolutionary history [14], and that the man-made design of exquisitely specific drugs is a tremendous undertaking.

A common problem encountered by modellers of chemogenomics data (large repositories of compound-target associations) that is equally a common concern for reviewers of such modelling exercises is the extreme sparseness of the compound-target matrix. The nature of compound screening in drug discovery brings with it that often many structurally similar compounds are tested against the same target, or target family, to identify structural determinants of activity and selectivity. This results in disproportionately many data points for isolated proteins, whereas other proteins are relatively deprived of the honour of being probed to that extent [15]. Consequently, every single chemogenomics dataset, with few exceptions such as the BioPrint database from CEREP [16], is unbalanced and sparse. This is a severe drawback from a modelling perspective as most likely any number for false positives can be expected to be an overestimate. The dataset we used comprises 1,309 compounds and for 804 of these we had target annotations in our repository. These annotations covered a total of 4,428 distinct proteins (as identified by their UniProt primary accessions) in a total of 19,871 compound-target associations. Thus, merely 0.5% of the compound-target matrix that we base our studies on is populated. This extreme sparseness is sobering at best considering that we retrieved the annotations from one of the largest existing repositories of compound bioactivities. Conversely, it illustrates straightforwardly that there is ample space for novel discoveries.

### Target prediction from gene signatures

We used a simple nearest neighbour technique to predict targets of compounds. To that end, we correlated the transcriptional profile of a query compound to all other profiles and retained the three nearest neighbours, that is the compounds corresponding to the three highest correlations. The targets of the neighbours are the predicted targets, and we consider a prediction successful if the intersection of predicted and real targets is non-zero. The overall accuracy for a given signature is the fraction of successful predictions; see section 'Materials and methods' for details. Unless otherwise stated, accuracy refers to the accuracy obtained when only the first nearest neighbour is considered.

**Figure 1:**
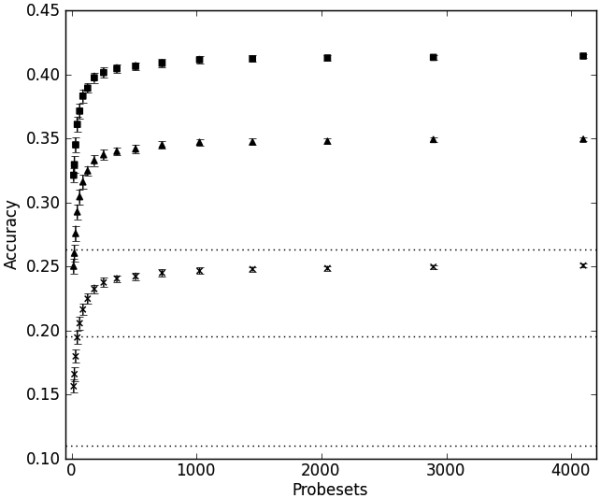
**Performance of random signatures levels off at around 300 probesets**. The dotted lines are the accuracies obtained (for increasing number of nearest neighbours from bottom to top) for randomly shuffled compound-target associations. This shows that although the signature probesets were selected randomly, they nonetheless yield a better target prediction accuracy than chance alone. One nearest neighbour: cross; two nearest neighbours: triangle; three nearest neighbours: square. Displayed data are the average accuracy values (*n *= 50) and the total length of the error bars is the corresponding standard deviation.

The term transcriptional signature is used for a subset of all probesets that is employed for the target predictions. Such signatures were derived using two data-driven methods: (1) based on all expression values; and (2) based on biological networks. For the latter part, we used all human interactions of the StringDB interaction database [17]. We retained the top-ranking 300 probesets for each of the selection methods described in section 'Methods and materials'. This cutoff was chosen as even for randomly selected signatures there was no increase in performance with more probesets (see next paragraph and Figure 1).

To establish a baseline for all further experiments, we determined the accuracy of guessing by using randomly shuffled compound-target associations. The accuracy obtained in this way ranges between 0.11 for one nearest neighbour and 0.26 for three nearest neighbours. It is interesting to note that even randomly selected probesets perform better than pure chance, for example with one nearest neighbour 0.11 versus 0.16 (Figure 1).

### Designed signatures

**Figure 2:**
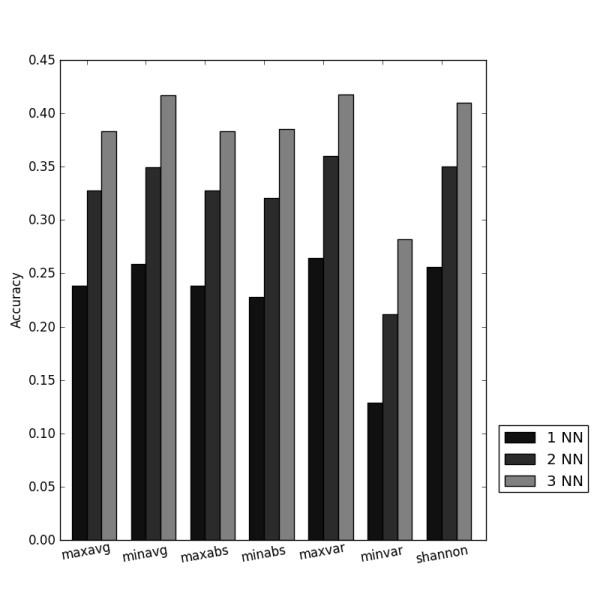
**Performance of the signatures derived from expression data for one, two or three nearest neighbours (NN)**. maxavg: highest mean expression; minavg: lowest mean expression; maxvar: highest standard deviation; minvar: lowest standard deviation; maxabs: highest mean of absolute expression value; minabs: lowest mean of absolute expression value and shannon: Shannon entropy of binned expression values.

We used two different groups of signatures for our experiments: one group was derived from the expression data itself, the other from biological interaction networks. Regardless of how the signatures were obtained, none produced an accuracy above 0.27. All signatures that were derived using expression data had accuracies in the range of 0.13 (for the minimum variance signature) to 0.26 (for the maximum variance signature, see Figure 2). Even with three nearest neighbours, the minimum variance signature was clearly the worst (Figure 2). The signature most different from all others consisted of the minimum variance probesets. This was consistent with what would be expected, as the genes corresponding to these probesets simply were not very responsive to perturbation. It is interesting to note that the genes that had the highest average expression were not very predictive; on the contrary, the signature comprised of the probesets that had the lowest average expression performed better. Consistent with the previous observations was that the probesets with the highest overall variance of expression were most useful for target prediction.

**Figure 3:**
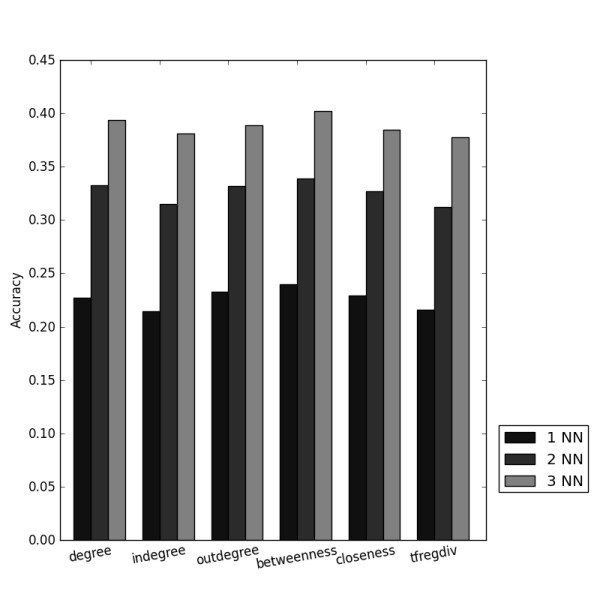
**Performance of the signatures derived from biological networks for one, two or three nearest neighbours (NN)**. betweenness: betweenness centrality; closeness: closeness centrality; degree: degree centrality; in-degree: in-degree centrality; out-degree: out-degree centrality; tfregdiv: diverse set of genes that are downstream of regulators of gene expression.

The signatures derived from biological networks all performed equally with accuracies around 0.23; all of them improved in a similar way with increasing numbers of nearest neighbours (Figure 3). The signature that performed best was based on the betweenness centrality of network nodes. This centrality is related to the number of shortest paths that go through a node. None of these signatures performed better than any signature derived from expression data.

### Genetically optimised signatures

**Figure 4:**
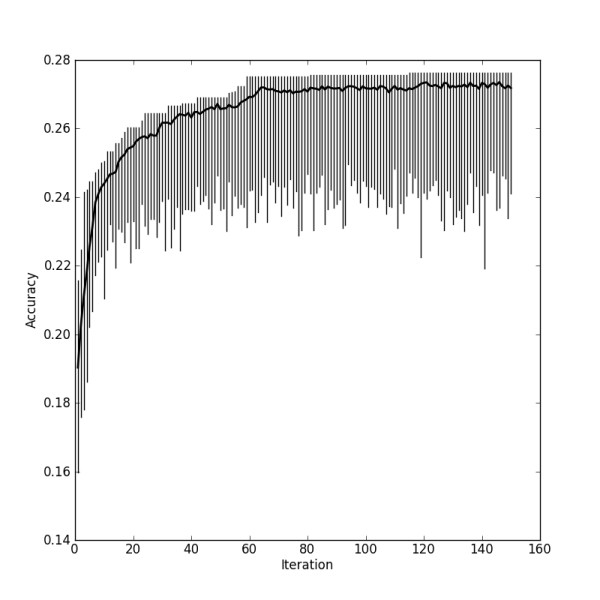
**Results of optimisation by a genetic algorithm of the signature with 64 probesets**. The vertical line for each iteration spans the range from worst to best fitness. The solid line indicates the mean fitness of all individuals in any iteration.

We used a genetic algorithm to evolve pools of 200 randomly initialised signatures for 150 generations. This resulted in an optimised set of genes for each signature size. Figure 4 shows the distribution of fitness scores over the range of the entire optimisation of 150 generations for a signature of 64 probesets. The decrease in the rate of improvement of the maximum fitness indicates that the genetic algorithm is close to converging to an optimal solution. Whereas there is no guarantee that it will ever be reached [18], Figure 4 shows that we are presumably very close to the maximally achievable accuracy for that signature size.

Overall, all of the genetically optimised signatures achieved accuracies above 0.26. Therefore, the smallest optimised signature with 32 probesets outperformed many of the expression-based signatures and also all network-based signatures. The signature that performed best contained 128 probesets and achieved an accuracy just below 0.30.

An analysis of the overlap of selected probesets between all of the optimised signatures revealed that very few probesets are shared. The highest overlap is achieved between the two largest signatures with 136 shared probesets between the signatures with sizes 1,448 and 2,048. The maximum overlap between two signatures is equal to the size of the smaller signature. Therefore, overlaps are expressed here as the fraction of the smaller signature that is common to the larger signature. The largest fractional overlap is between the signatures of sizes 256 and 2,048: 37 probesets (14%) of the smaller signature are found in the larger signature.

Even the smallest genetically optimised signature (32 probesets) performed basically equally well as the best performing signature derived from expression values (the 300 most variable probesets). Each of the 32 probesets of the smaller signature therefore seems to capture at least 10% more information than the 300 probesets of the larger signature. It can also be noted that these two signatures only share one probeset. The smaller, optimised signature is therefore not merely a result of the genetic algorithm choosing the most variable probesets.

**Figure 5:**
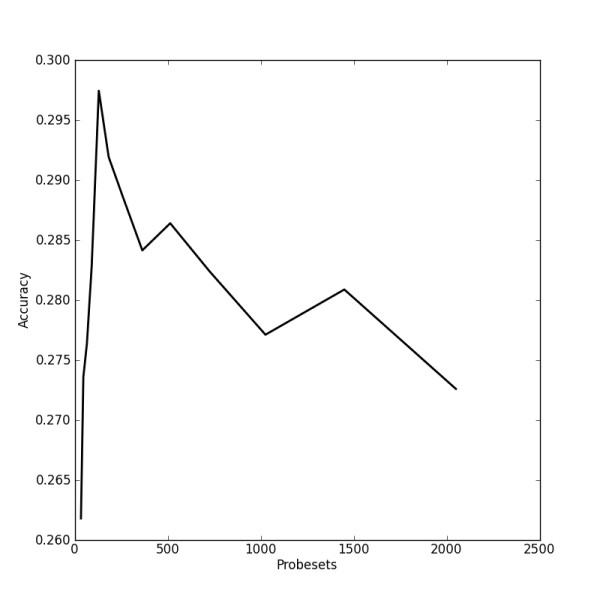
**Accuracies of signatures after 150 rounds of evolution by a genetic algorithm**. Maximum accuracy is achieved by a signature size of 128 probesets.

The good performance of very small, optimised signatures as well as the trend seen in Figure 5 indicates that larger signatures do not help in target prediction using our approach. Contrarily, they seem to add noise that is detrimental to performance. Obviously, such a trend might not be observed for other target prediction approaches such as reverse causal reasoning [19] where a larger signature might indeed provide more information to seed the reasoning algorithms.

### Analysis of gene signatures

We analysed whether the signatures derived by data-driven processes or the genetic algorithm are representative of any major biological processes. To that end, we calculated pathway enrichments for the designed signatures and the best-performing optimised signature with 128 probesets.

Overall, the signatures of the genes that have the highest absolute and highest mean expression yielded the most significant enrichments. The two most significantly enriched pathways were oxidative phosphorylation (-log*p *~ 100) and ubiquinone metabolism (-log*p *~ 58). The *p*-values increase rapidly to 10^-5 ^within the top five ranked pathways. The best-performing expression-based signature is enriched for cytoskeleton remodelling, regulation of cell cycle checkpoint G_1_/S and regulation of cellular metabolism (-log*p *values between 10 and 8).

The only other very significant enrichments were obtained with the network-derived signature based on the betweenness centrality of nodes. The enriched pathways were involved in protein folding (*p *~ 10^-19^) and regulation of G_1_/S transition (*p *~ 10^-18^). Noteworthy enrichments were also found for the signatures based on the degree centrality of nodes in the interaction network. All three of these signatures (degree, in-degree and out-degree) yielded several highly enriched pathways for nucleotide metabolism (10^-20 ^<*p *< 10^-17^). The results of all the enrichment calculations are provided as an Excel spreadsheet, see additional file 1.

The best-performing optimised gene signature with 128 genes showed a similar result as the one obtained for the highest absolute and highest mean expression signatures: oxidative phosphorylation (10^-113 ^<*p *< 10^-76^) and ubiquinone metabolism (*p *~ 10^-64^) were consistently the most significant pathways across several of the optimised signatures from different runs of the genetic algorithm.

The low *p*-values for oxidative phosphorylation are due to the large size of this pathway compared to all other pathways. This pathway contains several large complexes of the respiratory chain (mammalian complexes 1 to 4 and ATP synthase) and is composed of a total of 105 proteins. The ubiquinone metabolism pathway counts 74 proteins, 46 of which pertain again to mammalian complex 1. The constituents of the oxidative phosphorylation pathway, especially so the parts of the electron transport chain composed of complexes 1 to 4, are highly expressed in the mitochondria of all cells. Furthermore, rapidly dividing cancerous cells in culture, such as the ones used to derive the expression values used in this study, also require a lot of energy; thus, high expression levels are to be expected for members of the respiratory chain. Naturally, such highly expressed genes were selected for inclusion into the signature of genes with highest expression, which in turn explains the observed enrichment.

A more intriguing fact is that the same enrichment is observed for the best-performing optimised signature. This suggests that there is at least some overlap in the functionality of the genes of the two signatures: given the large size of the mammalian complexes 1 to 4 there need not be an overlap of the same genes, but in genes that belong to the same complex. The optimised signature therefore contains a significant part of genes that have a high level of expression overall, whereas the other genes were selected by the algorithm for other reasons. These other reasons are likely to remain unfounded due to the inherent lack of interpretability of results obtained from genetic optimisations, and we do not intend to speculate about those reasons at this point and leave this for further study. We note, however, that the enrichments obtained for the optimised signature are fundamentally different from and much more significant than those for an equal number of randomly selected probesets (data not included).

## Conclusion

We established a baseline for achievable target prediction accuracy using a simple 'guilt-by-association' method based on correlation of transcriptional profiles. The main objective of this study, however, is not target prediction *per se *but an investigation about how this can be achieved with gene signatures of varying nature and length. Two distinct groups of transcriptional signatures—expression data driven and based on biological interaction networks—were analysed for their performance; no striking differences between these groups were found. The optimisation of transcriptional signatures by a genetic algorithm led to the best-performing signatures and indicated that a maximum size of approximately 128 probesets is optimal. A signature of this size therefore extracted a maximum of biological variation of the investigated cellular systems. The genes of this optimised signature were predominantly found in pathways relating to oxidative phosphorylation and ubiquinone metabolism; this indicated that these biological processes might be the most generic way to capture compound perturbation of cells. We furthermore showed that it is possible to optimise very small signatures (32 probesets) for a particular purpose. Given that both groups of signatures—expression-based and network-based—perform similarly it is to be expected that a combination of both can lead to better signatures.

## Methods and materials

### Expression data and compound annotations

Our analyses are based on gene expression data from the Broad Institute's Connectivity Map 2 (CMAP2) [7]. Several cell lines were treated with a total of 1,309 different compounds and whole-genome expression levels were determined using Affymetrix gene chips. The cell lines with most measurements in CMAP2 were the human breast epithelial adenocarcinoma cell line MCF7, the prostate adenocarcinoma cell line PC3 and the human promyelocytic leukaemia cell line HL60. Expression levels were measured using the human Affymetrix chips (HT)HG-U133A [9]. The compounds were tested in batches with replicates, resulting in a total of 6,100 experiments. The combination of a compound, applied concentration, cell line and microarray platform used is referred to as a treatment instance.

We used a total of 22,267 probesets that were present in all treatment instances. CMAP2 data were downloaded from the Broad Institute's website and processed in *R *[20] using the *affy *[21] package. Robust multichip average [22] expression values were calculated for each treatment instance, and the expression values of each batch containing more than five treatment instances were then mean-centred on a probeset level using the average expression of each probeset in the corresponding batch [12]. In other words, the expression values we used correspond to expression after treatment relative to average expression in the batch; expression of vehicle (DMSO) treated cells does not enter the process. This procedure, originally proposed by Iskar et al. [12], has been found to be suitable for the elimination of batch effects for purposes very similar to ours.

The targets of any compound used in CMAP2 were obtained from an in-house bioactivity repository that comprises information both proprietary to Novartis and public such as ChEMBL and DrugBank [23,24]. We retained all targets of a compound at which it had an IC_50 _or *K_i _*value of ≤ 5 μM.

### Target prediction and accuracy measure

We determined nearest neighbours for each treatment instance by searching for treatments with highly correlated (Pearson product-moment correlation coefficient) gene signatures. Because the same molecule might have been tested several times under slightly different conditions (for example varying concentration, different cell line, different array platform), the nearest neighbour search was implemented in a way that prohibits it from finding a variation of a molecule as a neighbour for that molecule. The accuracies obtained would be higher without this restriction, but this would overestimate the true value that can be achieved in a real-world setting: in terms of target prediction the knowledge gained from a self-match is zero. We determined a maximum of three nearest neighbours for each treatment instance.

All of our analyses were assessed using the accuracy of target prediction, that is the fraction of all predictions that are considered successful. We considered a target prediction successful if the intersection of the target sets of query and nearest neighbour(s) is not empty. The main reason for this measure is the sparseness of compound-target annotations: any other measure would result in misleadingly low performance measures due to the large number of false positives/negatives; however, many of those predictions could actually be true if a complete compound-target matrix were available. An equally important factor for such a performance metric is the fact that in our setting all predicted targets have an equal rank. This is in contrast to other methods that provide a ranked list of targets. In separate experiments (not included here) we also used the *F*-measure, a weighted average of positive recall and positive precision that can be tuned to favour either recall or precision. The reliance on accuracy alone provides a realistic assessment of an achievable baseline for target prediction. Nevertheless, for certain applications it might indeed be worth to use other performance measures, for example to find a signature that minimises false negatives. For the precision of target prediction for the designed signatures, please refer to additional file 2.

The correlation calculations and nearest neighbour algorithms were implemented as a Python module using cython and CUDA on an NVIDIA GPU Tesla M2050 with 448 cores. This resulted in a speedup of more than two orders of magnitude compared to a single CPU implementation. The speed of this implementation was essential to get results from the genetic algorithm procedure in a reasonable amount of time. The source code used for any of the calculations is available from the authors upon request.

### Signature selection

In addition to designed signatures, we used signatures that were made up of randomly selected probesets to estimate the improvement that can be achieved when designed signatures are employed. We used 17 signatures containing 16 to 4,096 probesets in half-logarithmic steps in base 2. The signature sizes used were thus 16, 22, 32, 45, 64, 90, 128, 181, 256, 362, 512, 724, 1024, 1448, 2048, 2896, 4096. We randomly sampled 50 different signatures for each signature size; the reported accuracies for these signatures are therefore sample averages.

For expression-based signatures, the probesets were ranked according to the following criteria determined across all expression arrays in CMAP2: (1) highest mean expression; (2) lowest mean expression; (3) highest standard deviation; (4) lowest standard deviation; (5) highest mean of absolute expression value; (6) lowest mean of absolute expression value and (7) Shannon entropy of binned expression values; expression values were binned into 200 bins in the range [-5, 8].

For network-based signatures, we used the following criteria to score network nodes: (1) betweenness centrality; (2) closeness centrality; (3) degree centrality; (4) in-degree centrality; (5) out-degree centrality; (6) maximum average distance to reachable transcriptional modifiers.

The motivation for the last signature was to have a diverse set of genes that are downstream of regulators of gene expression. We first identified all regulators of gene expression (regulatory nodes) as any node in StringDB [17] that has at least one outgoing edge of mode 'expression'. For all nodes downstream of any regulatory node we then determined the average shortest path length to all reachable upstream regulators. Overall, this results in a total of 13 designed signatures.

### Optimisation with genetic algorithm

We used a genetic algorithm to determine an optimal signature for a given number of probesets. A population of 200 randomly initialised signatures was evolved for 150 generations. The objective function maximised by the genetic algorithm is the accuracy of prediction as defined above. The top 20% of each iteration were included for any subsequent iteration (elitism), the remaining 80% were obtained through crossover and mutation operations (crossover rate 70%, mutation rate 30%). Genetically optimised signatures were derived for the following signature sizes: 32, 45, 64, 90, 128, 181, 256, 362, 512, 724, 1024, 1448, 2048. The genetic algorithm was based on an example in 'Programming collective intelligence' [25].

### Pathway enrichments

We used GeneGO (now part of Thomson Reuters) Metacore to calculate pathway enrichments. This calculation is based on a hypergeometric null distribution for the intersection of the query set of genes and any given pathway [26]. The *p*-value corresponds to the probability of an intersection equal or greater to the observed one. This procedure is equal to a Fisher's exact test.

## Abbreviations

CMAP: Broad Institute's Connectivity Map, build 02; GPU: graphics processing unit; MoA: mechanism of action.

## Competing interests

The authors declare that they have no competing interests.

## Supplementary Material

Additional file 1 **Pathway enrichment for the designed gene signatures**. Pathway enrichment for the designed gene signatures. Click here for file

Additional file 2 **Precision of prediction for designed signatures**. Excel spreadsheet with precision of prediction for designed signatures. Click here for file
